# In Vitro and In Vivo Comparisons of Activated Charcoal and Biochar as Dietary Treatments for Controlling Boar Taint

**DOI:** 10.3390/biom15091257

**Published:** 2025-08-30

**Authors:** Melissa Parent, Christine Bone, Lee-Anne Huber, E. James Squires

**Affiliations:** Department of Animal Biosciences, University of Guelph, Guelph, ON N1G2W1, Canada

**Keywords:** boar taint, biochar, activated charcoal, spent filter aid, binding agents, androstenone, skatole, estrogens, enterohepatic circulation

## Abstract

Activated charcoal (AC) is an adsorbent that can prevent the accumulation of boar taint-causing compounds in the fat, but is not an approved dietary additive for livestock animals. Biochar (BC) is a similar feed-approved charcoal adsorbent that may be an alternative dietary additive to control boar taint. This study was conducted to evaluate AC and BC, both in vitro and in vivo, as dietary treatments for boar taint. This was done by first conducting an in vitro binding study to compare binding between AC, BC, and spent filter aid (SFA) for boar taint compounds. Results of the in vitro study showed that both AC and BC had significantly higher *B_max_* for androstenone (AC: 97.2 ± 0.4% and BC: 84.5 ± 0.8%) and skatole (AC: 106.1 ± 0.2%, BC: 113.2 ± 0.7%), compared to SFA with a *B_max_* of 50.5 ± 0.2% for androstenone and 97.1 ± 5.3% for skatole. AC and BC were then tested as feed additives in finisher diets fed to slaughter weight boars. Both adsorbents were successful at preventing boar taint in a subset of animals (83%), while having no effect on plasma levels of estrone sulfate or androstenone, and growth and performance parameters. These findings suggest that BC is a suitable alternative for AC as a dietary additive to prevent boar taint.

## 1. Introduction

Entire male pigs can develop a meat quality issue known as boar taint, which is characterized by a urine- or feces-like odour that develops in heated pork products in meat from these animals. Boar taint is caused primarily by the accumulation of two compounds, androstenone (5α-androst-16-en-3-one) and skatole (3-methyl indole) in the fat. Skatole is synthesized through the breakdown of tryptophan by microbes in the gastrointestinal tract [1], while androstenone is a sex steroid that is produced in the testis [2].

The enterohepatic circulation (EHC) describes the movement of compounds between the liver and the gut [3]. In the EHC, compounds first undergo metabolism in the liver and their conjugated metabolites are then incorporated into the bile and released into the small intestine [3,4,5]. Gut microbes can act to deconjugate the compounds, returning them to their active form that can be reabsorbed into the systemic circulation and transported back to the liver via the hepatic portal vein [6]. The EHC is well established to modulate the concentrations of estrogens in the body [7,8], with E1S (estrone-1-sulfate) being the most abundant estrogen produced by boars [9]. Since estrogens and compounds responsible for boar taint share structural and hepatic metabolism similarities [7,10], it has been suggested that the EHC may also influence circulating levels of androstenone [11]. Therefore, disruption of the EHC may be an effective method to reduce levels of androstenone to prevent the development of boar taint. Binding of skatole in the gut may also decrease its absorption from the gut and increase its excretion in the feces.

Activated charcoal (AC) is a binding agent that has been used as a dietary treatment to disrupt the EHC [12]. Its initial use was in human and animal medicine to bind toxins and other unwanted compounds in the gastrointestinal tract and prevent their absorption into the systemic circulation [13]. The characteristic that makes AC so versatile and adsorptive is the activation process that produces pores and increases its surface area [14]. A preliminary feeding trial using a 5% AC-supplemented diet found that dietary AC treatment was effective for reducing androstenone levels in the fat of slaughter weight boars [11]. Based on this, AC was suggested to bind androstenone metabolites that have been excreted from the liver to the intestines, preventing their reabsorption into systemic circulation and accumulation in fat. While AC is an effective binding agent, its high price point and lack of approval for use in livestock feed limit its use in the swine industry [15]. Park & Squires [16] tested a variety of mineral-based adsorbents including bentonite, diatomaceous earth, spent filter aid (SFA), which is a byproduct of corn syrup production containing one-third diatomaceous earth, and Jumpstart 360 (hydrated sodium calcium aluminosilicate). Of these adsorbents, SFA showed the most promise as it was able to bind both androstenone and skatole in vitro; however, it was not able to bind androstenone to the same degree as AC [16]. As a niche byproduct, there is limited information available on the binding characteristics, physical properties, and absorptive behaviour of SFA.

Biochar (BC) is a charcoal-based binding agent that has been used in the agricultural sector for soil remediation and as a dietary additive for reducing methane emissions in cattle [17,18]. It can be produced from any form of biomass that has undergone pyrolysis, and it differs from AC in that it is not activated [14]. BC possesses high porosity and a large surface area, both of which contribute to its high pore-surface area ratio [17]. Additionally, BC has been shown to catalyze redox reactions via storage and donation of electrons [17]. Since BC is approved for use in livestock feed and is cheaper than AC, it could be an effective alternative to AC that could be quickly and easily implemented into a swine production setting. Therefore, this study characterized the in vitro binding kinetics of BC for androstenone, skatole, and estrogens relative to SFA and AC, and evaluated both AC and BC as dietary treatments at 5% inclusion to determine whether BC is a suitable alternative to AC for controlling boar taint.

## 2. Materials and Methods

### 2.1. In Vitro Binding Studies

#### 2.1.1. Preparation of Adsorbent and Adsorbate Solutions

The adsorbents (binding agents) used for this study were AC (>95% carbon, <5% moisture, surface area 875 m^2^/g; NORIT A, Acros Organics, Fair Lawn, NJ, USA), BC (78.5% carbon, 44.5% moisture, wet weight, surface area 443 m^2^/g; Blue Rock Animal Nutrition, Innisfail, AB, Canada), and SFA (Ingredion Canada, London, ON, Canada). Binding agents were prepared in a phosphate-buffered saline (PBS; 139 mM NaCl, 8.5 mM Na_2_HPO_4_, 1.5 mM KH_2_PO_4_, 2.7 mM KCl, pH 7.4). The AC and BC solutions were diluted in PBS to a working concentration of 2 mg/mL and SFA was diluted to a working concentration of 80 mg/mL [16].

Adsorbate solutions, skatole (46 µM containing 6% ethanol, Sigma-Aldrich, Saint Louis, MO, USA), estrone (E1; 74 mM containing 10% ethanol, Steraloid Co., Wilton, NH, USA), E1S (57 mM containing 10% ethanol, Sigma-Aldrich, Saint Louis, MO, USA), and androstenone (20 µM containing 10% ethanol, Steraloid Co., Wilton, NH, USA) were made as concentrated stocks in ethanol and then diluted in PBS. The steroid solutions were then further diluted 1:1 (*v*/*v*) with radiolabelled steroid solutions with 15,000–18,000 CPM. The final concentrations of steroid solutions were [^3^H]-E1 (37 mM, 0.10 µCi/mmol, 5% ethanol, Perkin-Elmer, Boston, MA, USA), [^3^H]-E1S ammonium sulfate (28.5 mM, 0.15 µCi/mmol, 5% ethanol, Perkin-Elmer, Boston, MA, USA), and [^3^H]-androstenone (10 µM, 0.39 µCi/µmol, 5% ethanol, Moravek Biochemicals, Brea, CA, USA). The final concentration of skatole (23 µM) present in the incubation was selected to be comparable with the physiological concentration of skatole present in the cecum of nursery piglets [19]. Sex steroid concentrations used in this study were above those present in mature boars [3].

#### 2.1.2. Adsorption Assays

Adsorption assays were performed according to the methods previously described by Jen and Squires [20]. Briefly, AC and BC were diluted to concentrations of 1, 0.75, 0.50, 0.25, 0.13, 0.063, 0.031, 0.015 and 0.0078 mg/mL. SFA was diluted to concentrations of 40, 20, 10, 5, 2.5, 1.25, 0.63, 0.31 and 0.078 mg/mL consistent with the effective binding range for steroids and boar taint compounds reported by Park and Squires [16]. Each concentration of adsorbent was mixed with an equal volume of one of the radiolabelled steroid solutions, [^3^H]-E1, [^3^H]-E1S, and [^3^H]-androstenone (in triplicate) or skatole (in duplicate).

All test tubes were incubated at 37 °C for 30 min and then centrifuged at 2560× *g* for 30 min. Radioactivity in the supernatant was quantified through liquid scintillation counting with CytoScint scintillation fluid (MP Biomedicals, Solon, OH, USA). Skatole concentrations were assessed using high-performance liquid chromatography (HPLC) using a modified protocol previously described by Lanthier et al. [19]. Briefly, samples were diluted 80x with PBS, centrifuged for 10 min at 8000× *g*, filtered with 0.2 µm nylon syringe filter (Fisher Scientific, Toronto, ON, Canada), and then injected onto a C18 reverse-phase Luna, 5 µm C18 column (250 × 4.60 mm) (Phenomenex, Torrance, CA, USA). The solvent system consisted of buffer A: 10% acetonitrile, 90% 5mM KH_2_PO_4_ and buffer B: 100% acetonitrile. The HPLC procedure ran as follows: 0 min—50% A and 50% B, 8 min—20% A and 80% B, 8.1 min—100% B, 15 min—100% B, 15.1 min—50% A and 50% B, 20 min—50% A and 50% B. The elution of skatole occurred at 7 min and was detected through fluorescence with an excitation wavelength of 285 nm and an emission wavelength of 350 nm.

### 2.2. In Vivo Feeding Trial

#### 2.2.1. Animals and Experimental Design

All animals were housed and handled in accordance with the Animal Care Committee guidelines at the University of Guelph and met the requirements of the Canadian Counsel of Animal Care. Boars were housed at the Ponsonby Research Station at the University of Guelph in two blocks of twenty-five animals separated into two to three boars per pen. All animals had ad libitum access to feed and water.

**Figure 1:**
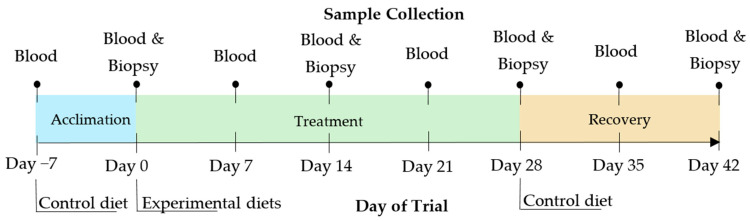
Experimental timeline depicting acclimation, treatment, and recovery duration as well as sample collection.

The trial design, dietary treatment inclusion levels, and sample analysis followed the methodology previously described by Jen and Squires [11], with animals slaughtered at weights typical for Canadian commercial swine production. Beginning at 71.31 ± 1.26 kg body weight and 103 ± 0.86 days of age (means ± standard error), two blocks, totalling fifty [(Yorkshire × Landrace) × Duroc] boars from Alliance Genetics Canada Inc. were introduced to the control diet for a one-week acclimation period. Boars were then randomly assigned to one of three experimental diets, either the control diet (*n* = 16), AC diet (*n* = 17), or BC diet (*n* = 17), totalling six pens per treatment (day 0). Boars remained on their respective treatment diets for four weeks, and then all animals were transitioned back to the control diets for a two-week recovery period, which allowed for evaluation of boar taint status in the absence of dietary treatments. Animals were slaughtered after the final sampling in the recovery period, first being rendered unconscious in a CO_2_ chamber, then exsanguinated. The experimental timeline is depicted in Figure 1. Due to the inability to sample the second block of boars at days 0 and 28, they remained on the acclimation diet and treatment diet for an additional four days each to ensure key sample time points were not missed.

Blood was collected in heparinized tubes (Fisher Scientific—Whitby, ON, Canada) from the orbital sinus on days −7, 0, 7, 14, 21, 28, 35, and 42. Backfat biopsies were collected bi-weekly (days 0, 14, 28, and 42) by disinfecting the area with 70% ethanol and using a biopsy punch to collect a sample of the fat on the back of the neck.

#### 2.2.2. Diet Compositions

Table 1 compares the composition of the control, 5% AC, and 5% BC diets. The control diet contained a 5% cellulose filler to ensure that the control diet and charcoal diets remained isoenergetic. This allowed for a comparison of growth parameters among animals on all diets. Otherwise, the diets were identical in terms of ingredient composition.

#### 2.2.3. Immunoassays and Skatole Analysis

Blood samples were centrifuged at 1789× *g*. Plasma was used to measure concentrations of E1S via radioimmunoassay (RIA), using a protocol adapted from Raeside et al., [21]. Androstenone concentrations were quantified in both the fat and plasma through an androstenone-specific enzyme-linked immunosorbent assay (ELISA) [21,22,23]. Briefly, fat samples were melted and extracted in methanol and incubated at −20 °C for one hour, centrifuged at 1789× *g*, and the methanol fraction was diluted 20× in PBS before being used in the ELISA. Plasma was used directly for ELISA without the need for extraction. Fat for skatole analysis was extracted by incubating the fat in methanol at −20 °C for one hour and using the methanol extract for the skatole HPLC analysis described above [24].

#### 2.2.4. Growth and Performance Parameters

Each boar was weighed prior to sample collection, and feed intake was recorded weekly on a per-pen basis. This allowed for the calculation of average daily gain (ADG) on a per-animal basis and feed conversion ratio (FCR) on a per-pen basis. Each of these measures was calculated for the entire trial (day −7 to 42), as well as during the treatment period from day 0 to 28, to determine any differences between the control and the charcoal treatment diets.

### 2.3. Statistical Analysis

The in vitro binding curves assessing the binding of adsorbates (androstenone, skatole, E1, and E1S) by adsorbents (AC, BC and SFA) were calculated by dividing the amount of adsorbate remaining by the initial adsorbate concentration added to each incubation and subtracting this from 100. The Solver tool in Microsoft Excel (2016) was used to assess the binding kinetics of the adsorbents and the data was then fit to a modified Michaelis-Menten equation, shown in Equation (1) [20]:

(1) $$B=\frac{\left(B_{max}\right)\left(C\right)}{K+C}$$

where *B* is the percentage of adsorbate bound by a given adsorbent; *B_max_* is the maximum binding achieved by a given binding agent expressed as a percentage; *C* represents the concentration of a given adsorbent (mg/mL) and *K* is the concentration of a given binding agent that binds 50% of *B_max_*.

The efficiency of binding was estimated as *B_max_/K*.

Statistical analysis was performed using SAS 9.4 (SAS Institute, Cary, NC, USA), with the Shapiro–Wilk test first used to assess and confirm the normality of the data. The results of the in vitro binding studies were evaluated using proc GLM procedure and the in vivo results were analysed with proc MIXED using a repeated measures ANOVA for hormone concentrations and a standard ANOVA for performance parameters. Main effects of dietary treatment and time, a random effect of block to account for the repetition of the study using two distinct groups of animals, and an interaction effect of treatment over time were assessed. A Tukey–Kramer post hoc adjustment was used in all analyses to account for multiple comparisons. A significance level of *p* < 0.05 was used for all analyses.

## 3. Results

### 3.1. In Vitro Adsorption Assays and Michaelis-Menten Binding Kinetics

**Figure 2:**
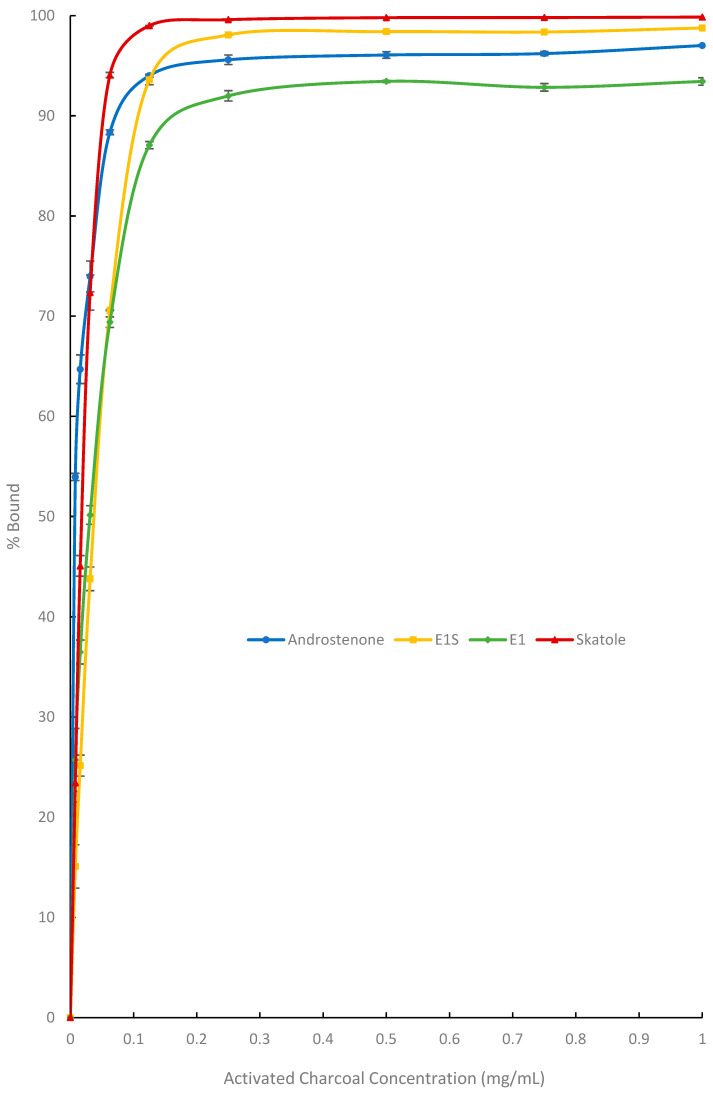
Percentage of activated charcoal (AC) that bound each adsorbent androstenone (triplicate), E1S (estrone-1-sulfate, triplicate), E1 (estrone, triplicate), and skatole (duplicate). Data are presented as means ± standard error.

**Figure 3:**
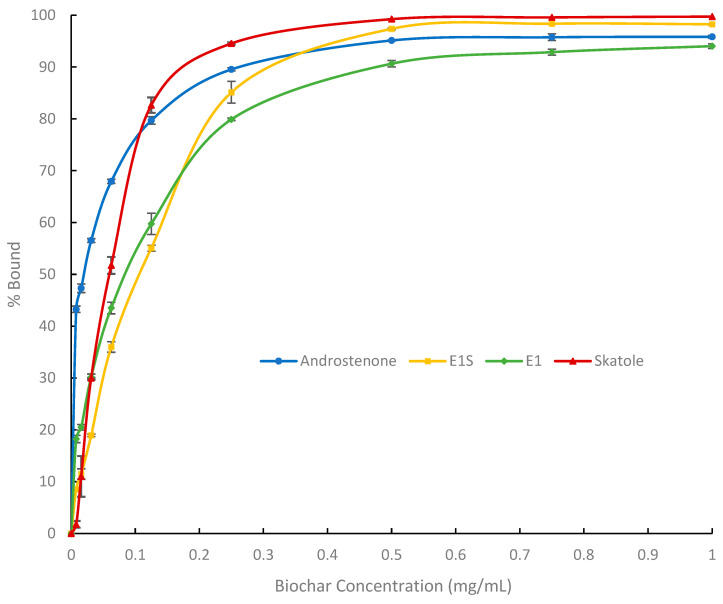
Percentage of biochar (BC) that bound each adsorbent androstenone (triplicate), E1S (estrone-1-sulfate, triplicate), E1 (estrone, triplicate), and skatole (duplicate). Data are presented as means ± standard error.

**Figure 4:**
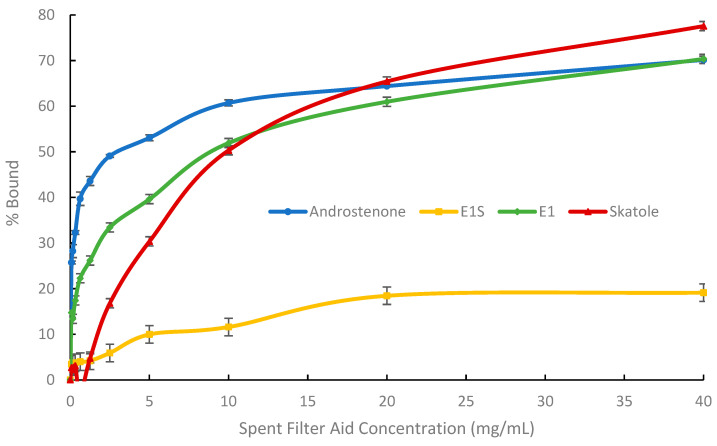
Percentage of spent filter aid (SFA) that bound each adsorbent androstenone (triplicate), E1S (estrone-1-sulfate, triplicate), E1 (estrone, triplicate), and skatole (duplicate). Data are presented as means ± standard error.

Binding curves (Figure 2, Figure 3 and Figure 4) were generated to visualize the percentage of radiolabelled steroid and skatole that was bound by each adsorbent. The binding kinetics of the three adsorbents were assessed based on their maximum binding (*B_max_*), *K* (concentration of binding agent to bind 50% *B_max_*) and *B_max_/K* (binding efficiency) and are shown in Table 2.

Figure 2 shows the ability of AC to bind E1, E1S, androstenone and skatole. Binding plateaus exceeded 95% for all compounds and the *B_max_* of AC for all four compounds was between 97.2% and 107.7% (Table 2). The binding of androstenone and skatole plateaued at a concentration of 0.13 mg/mL and 0.25 mg/mL, respectively, while the maximum binding of estrogens occurred at 0.50 mg/mL, demonstrating that adsorbate binding by AC was not specific for androstenone and skatole.

The binding results for BC (Figure 3) showed a similar response to AC where androstenone and skatole reached a plateau first, followed by estrogens. However, relative to AC, a greater concentration of BC was required to reach these plateaus. Androstenone, skatole and E1S plateaued at approximately 0.50 mg/mL, while the binding of E1 reached a plateau around 0.75 mg/mL of BC. As shown in Table 2, BC had a significantly lower *B_max_* for androstenone compared to AC (AC: 97.2 ± 0.4%, BC: 84.5 ± 0.8%; *p* < 0.0001). Whereas the *B_max_* for skatole was similar between AC (106.1 ± 0.2%) and BC (113.2 ± 0.7%). Additionally, *K* values for all adsorbates were not different between AC and BC.

Binding studies with SFA required a much higher concentration than both AC and BC. The binding of androstenone, skatole, and E1 did not reach a plateau at a concentration of 40 mg/mL, which was the highest concentration of SFA evaluated; for these adsorbates, the binding was less than 80% (Figure 4). The binding of E1S was the lowest, reaching 19.1% bound at a SFA concentration of 40 mg/mL, with a *B_max_* of 97.1 ± 5.3% (Table 2). The *K* value of SFA for E1S was similar to that of BC; however, for all other adsorbates, SFA had significantly lower *B_max_* and *B_max_/K* values, and significantly higher *K* values compared to both AC and BC (*p* < 0.0001).

### 3.2. In Vivo Animal Feeding Trial

#### 3.2.1. Classification of Boar Taint Status and Treatment Response

**Figure 5:**
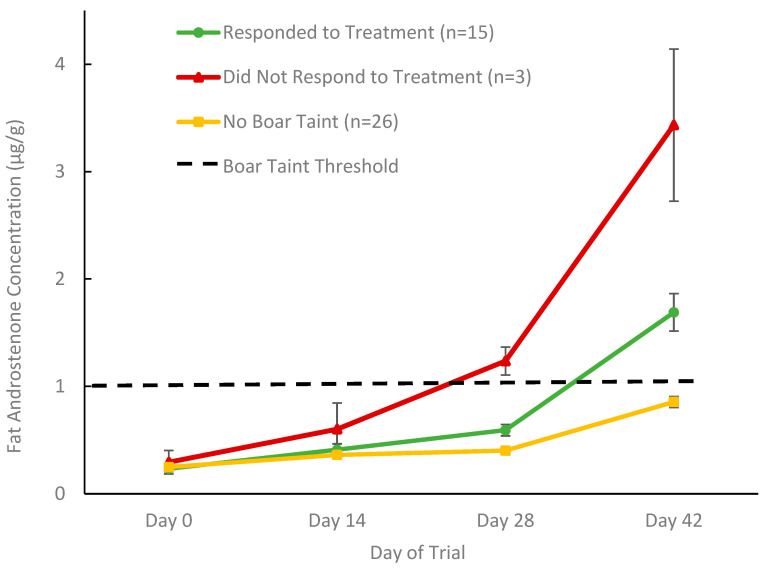
Fat androstenone concentrations (µg/g) from treatment and control boars, excluding control boars that developed boar taint, were used to determine phenotypes reflecting boar taint status and treatment response using a boar taint threshold of 1 µg/g. Data are presented as means ± standard error.

To compare the efficacy of BC and AC as dietary treatments for controlling boar taint, androstenone concentrations were assessed by ELISA in backfat biopsy samples collected at days 0, 14, 28, and 42 of the feeding trial with AC and BC. Since boar taint does not develop in every animal, boars were classified into three distinct phenotypes related to their boar taint status and treatment response (Figure 5), using an established boar taint threshold of 1 µg/g fat androstenone [11]. Animals whose fat androstenone concentrations never exceeded the 1 µg/g threshold by the end of the recovery period (day 28–42) were classified as not developing boar taint. Animals whose fat androstenone concentrations only exceeded the threshold during the recovery period (after day 28) were classified as responding positively to treatment, as they developed boar taint once the AC or BC charcoal treatment was removed. Finally, animals were classified as not responding to treatment if their fat androstenone concentrations exceeded the threshold before the recovery period (day 28), while they were still receiving dietary AC or BC charcoal treatment. Additionally, fat skatole concentrations evaluated by HPLC showed that all animals were below the threshold of 200 μg/g of skatole in the fat at all time points in the trial [25], so the effect of charcoal treatment on fat skatole could not be evaluated. Supplementary Table S1 provides a further overview of the animal feeding trial results, grouped by diet and by treatment response.

Based on these classifications, 52% of the boars did not develop boar taint (10 control, 8 AC, and 8 BC) and therefore did not require dietary AC or BC treatments (Figure 5). Of the animals that developed boar taint and received one of the AC or BC dietary treatments, 83% responded positively as their fat androstenone levels remained below the boar taint threshold until the recovery period, when the treatment was discontinued. The remaining 17% of animals (2 AC and 1 BC) developed boar taint while receiving either AC or BC dietary treatment.

#### 3.2.2. Fat Androstenone Concentrations in Treatment and Control Boars with Boar Taint

**Figure 6:**
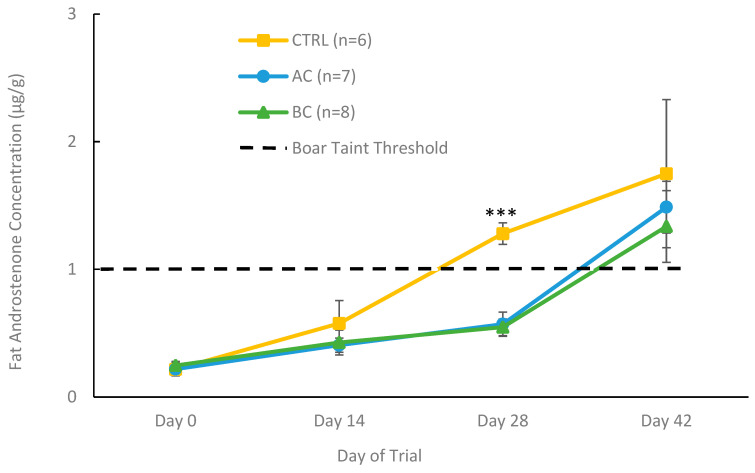
Fat androstenone concentrations (µg/g) of boars from day 0 to 42 receiving one of three experimental diets: control (CTRL), activated charcoal (AC), or biochar (BC). The control animals shown were all classified as having boar taint using a threshold of 1 µg/g, while all AC- and BC-treated boars were classified as having boar taint and responding to treatment. Data are presented as means ± standard error. Significance between treatment groups is indicated by *** (*p* < 0.0001).

To accurately compare the ability of AC and BC diets to prevent boar taint, analyses only included animals that responded positively to treatment, along with control animals that developed boar taint during the treatment period (on or before day 28). Figure 6 shows the fat androstenone concentrations across the trial in control boars that developed boar taint as well as AC-treated, and BC-treated boars that developed boar taint and responded to treatment. All animals from day 0 to 14 had fat androstenone levels below the boar taint threshold, indicating they did not have boar taint at these time points. On the last day of the treatment period (day 28) control boars that developed boar taint had a mean fat androstenone concentration of 1.28 ± 0.084 μg/g, while boars that responded to BC (0.55 ± 0.065 μg/g) and AC (0.57 ± 0.095 μg/g) had lower fat androstenone concentrations (*p* < 0.0001) than the control, which were well below the boar taint threshold of 1 μg/g. In the recovery period (day 42), all animals that responded to one of the charcoal treatment diets and had fat androstenone concentrations below the boar taint threshold on day 28, had developed boar taint and their fat androstenone concentrations were no longer different from the control boars.

#### 3.2.3. Plasma Hormone Concentrations in Treatment and Control Boars with Boar Taint

**Figure 7:**
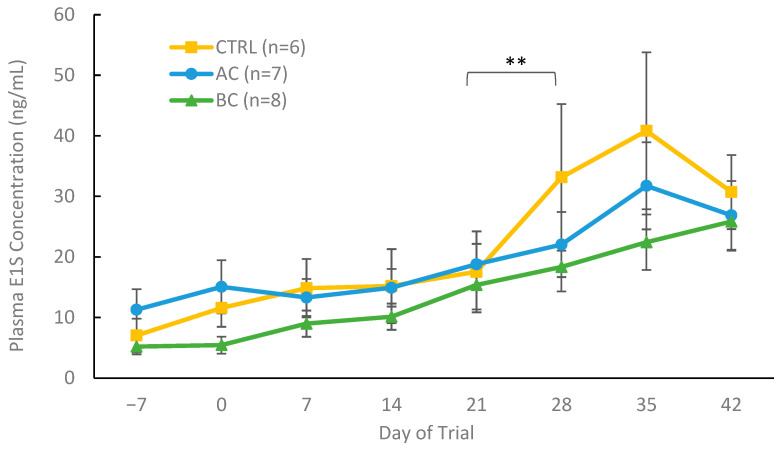
Plasma E1S concentrations (ng/mL) of boars from day 0 to 42 receiving one of three experimental diets: control (CTRL), activated charcoal (AC), or biochar (BC). The control animals shown were all classified as having boar taint using a threshold of 1 µg/g, while all AC- and BC-treated boars were classified as having boar taint and responding to treatment. Data are presented as means ± standard error. Significance within each treatment group over time is indicated by ** (*p* = 0.005). No significant differences (*p* > 0.05) were observed between the control, BC, and AC groups at any of the assessed time points.

Plasma E1S concentrations are indicative of the growth and sexual maturation of boars, and since our in vitro binding studies demonstrated that AC and BC readily bind estrogens, we quantified plasma E1S levels throughout the trial using an RIA. Control-fed, AC- and BC-treated boars had similar plasma E1S values at each time point throughout the treatment period of the trial, shown in Figure 7. Plasma E1S levels increased (*p* = 0.005) between days 21 and 28 for control animals, but not BC- and AC-treated boars. There was a significant increase in plasma E1S values when comparing the levels at day −7 versus day 42 in BC-treated boars (*p* = 0.0003), whereas E1S concentrations in AC-treated boars increased numerically, but not significantly. This plasma E1S profile is in line with animals reaching puberty and increasing the production of estrogens to help initiate sexual development [9,26]. The similarity in plasma E1S levels between control-fed, AC and BC treatments during the treatment period (days 0 to 28) suggests that both charcoal adsorbents did not alter the circulating homeostatic levels of estrogens in charcoal-treated boars.

**Figure 8:**
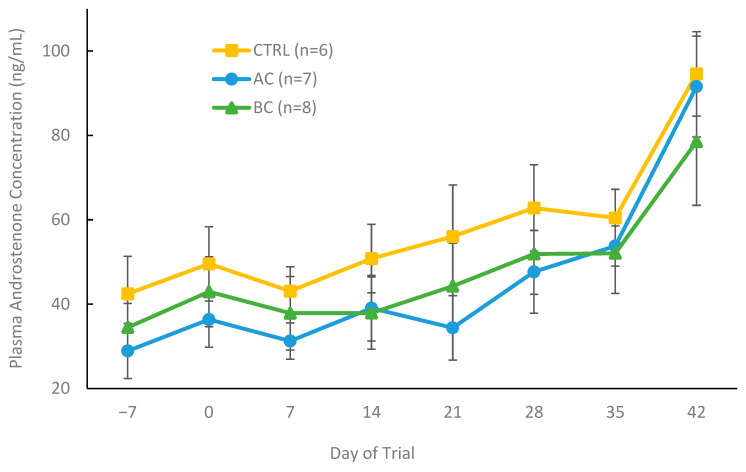
Plasma androstenone concentrations (ng/mL) of boars from day 0 to 42 receiving one of three experimental diets: control (CTRL), activated charcoal (AC), or biochar (BC). The control animals shown were all classified as having boar taint using a threshold of 1 µg/g, while all AC- and BC-treated boars were classified as having boar taint and responding to treatment. Data are presented as means ± standard error. No significant differences (*p* > 0.05) were observed between the control, BC, and AC groups at any of the assessed time points.

While plasma androstenone concentrations are not directly indicative of boar taint status or fat androstenone levels, they can reflect the balance between the rate of synthesis and metabolism of androstenone [9,27]. Therefore, we quantified plasma androstenone concentrations throughout the trial in both control and treatment animals by ELISA. Figure 8 shows the mean plasma androstenone concentrations in AC- and BC-treated boars that developed boar taint and responded to treatment, along with control boars that developed boar taint, based on the classification criteria described above. Plasma androstenone levels increased numerically but not significantly each week as the trial progressed. Plasma androstenone concentrations did not differ among control, AC, and BC-treated boars at any time point in the trial.

#### 3.2.4. Animal Performance Parameters

To evaluate the effect of dietary treatments on growth performance, animal weight, ADG, and FCR were calculated across the trial and compared to those of control-fed boars. The growth performance parameters for both treatment and control groups are shown in Table 3. ADG and FCR were calculated over the entire trial as well as during the treatment period alone to better isolate the effects of the dietary charcoal treatments. There were no differences in body weight, ADG or FCR among the control, AC-, or BC-treated groups at any time point in the trial. This indicates that a 5% inclusion of BC and AC did not negatively affect the nutrient availability of either treatment diet, and all boars were able to grow and develop similarly, regardless of diet.

## 4. Discussion

The charcoal-based adsorbent, AC, and the mineral-based adsorbent, SFA, were previously studied as possible dietary adsorbents for the disruption of the EHC and prevention of boar taint [11,16]. The AC was effective at controlling boar taint but is not an approved feed additive for livestock, while SFA is safe for consumption but does not bind androstenone as effectively as AC [11,15,16]. In this study, we investigated BC, a charcoal-based adsorbent similar to AC and an approved livestock feed additive, as a dietary binding agent for controlling boar taint. Specifically, we evaluated the in vitro binding affinity of BC for androstenone, skatole, and estrogens, compared to the previously tested adsorbents AC and SFA. We then conducted animal feeding trials to assess whether BC could serve as an effective alternative to AC as a dietary additive for controlling boar taint in slaughter-weight boars.

### 4.1. In Vitro Binding Affinity of Binding Agents for Estrogens and Boar Taint-Causing Compounds

Using a modified Michaelis-Menten equation, we evaluated the maximum binding (*B_max_*), binding affinity (*K*), and binding efficiency (*B_max_/K*) of AC, BC, and SFA for boar taint-causing compounds and estrogens in vitro. The *B_max_* and *K* values were similar between AC and BC for all adsorbates, except for the *B_max_* for androstenone, which was slightly greater for AC than BC (97% vs. 85%) and the *B_max_* for E1S, which was greater for BC (108% vs. 117%). However, the binding efficiency (*B_max_/K*) of AC was 1.8 to 4.6 times greater than that of BC for the different adsorbates.

Consistent with these results, Appell et al. [28] previously reported similar binding affinities between AC and three types of BC (pine, olive wood, and horticulture) for zearalenone, a mycotoxin similar in structure to estrogens such as E1 and E1S. In contrast, mineral-based adsorbents such as bentonite and montmorillonite were significantly less effective at binding mycotoxins compared to charcoal-based adsorbents [28]. Additionally, Park and Squires [16] demonstrated that SFA binds E1, E1S, and androstenone less efficiently than AC. In the present study, the *B_max_* and *B_max_/K* of SFA for androstenone, skatole, E1, and E1S were significantly lower than those of both BC and AC. These results demonstrate that BC and AC are comparable in their ability to bind boar taint-causing compounds and estrogens in vitro, with BC needing a slightly larger inclusion rate to achieve these results, likely due to its lower carbon content and smaller surface area compared to AC. In contrast, SFA preferentially binds skatole over estrogens and androstenone, and is significantly less efficient at binding all adsorbates. This may be due to differences in relative polarity or surface level interactions of the four adsorbates, but additional work in this area is needed. This suggests that BC and AC may have similar efficacy as dietary binding agents for controlling boar taint; however, their inability to selectively bind boar taint compounds without also binding estrogens could have negative effects on the animal’s growth and sexual development. To assess this, BC and AC were further evaluated as dietary binding agents for their ability to prevent boar taint in vivo, as well as their potential effects on growth performance.

### 4.2. Using AC and BC In Vivo as a Dietary Control for Boar Taint

To compare BC and AC as feed additives for controlling boar taint, fat androstenone levels were evaluated in boars fed either a 5% BC-supplemented diet, a 5% AC-supplemented diet, or a control diet containing 5% cellulose filler for 28 days, followed by a two-week recovery period where the treatment diets were discontinued. The recovery period was used to assess whether boars had the capacity to develop boar taint, as it is a complex issue and not all boars develop it or respond to treatment in the same way. To the best of our knowledge, this is the first study to consider boar taint status when evaluating the efficacy of dietary treatment strategies, which provides a more accurate framework for assessing treatment response. Interestingly, more than 50% of the fifty boars in our study did not develop boar taint. Of the animals that developed boar taint, AC and BC diets were able to maintain androstenone levels below the boar taint threshold of 1 µg/g in 83% of the animals. This left one boar receiving the BC diet and two boars receiving the AC diet that still developed boar taint during the treatment period. These results support those of a prior study by Jen & Squires [11], in which dietary AC successfully reduced fat androstenone levels below the boar taint threshold during the treatment period of thirteen Yorkshire boars. Genetic improvements, through selective breeding and the use of crossbred boars, may be contributing to the reduction of boar taint. Additionally, since the development of boar taint is dependent on many different physiological, genetic, nutritional, and environmental factors, it is possible that the EHC may be more influential in animals that responded to the charcoal-based treatments compared to those that did not [29]. Taken together, our results demonstrate that BC can prevent boar taint in a subset of slaughter weight boars with similar efficacy as AC, and highlight the importance of classifying animals by their boar taint status and treatment response when evaluating dietary treatments for the prevention of boar taint. Given these findings, future studies should aim to identify biological and genetic markers that are associated with boar taint development and treatment responsiveness. Additionally, biochar should be evaluated alongside fermentable carbohydrates, another promising dietary approach for controlling boar taint [30,31,32,33], as these treatments may act through different mechanisms and could be effective in distinct subsets of animals.

Plasma concentrations of androstenone and E1S were also measured throughout the trial to compare the effects of the BC and AC diets on circulating levels of these compounds. At all sampling time points during the treatment period, there were no differences in plasma androstenone or E1S levels between control animals and animals receiving BC or AC diets. Plasma concentrations of steroid hormones are influenced by many factors, which may help explain the lack of observed differences. Additionally, it is well established that circulating levels of steroid hormones depend on the balance between their rates of synthesis and metabolism [34,35]. In boars, estrogens are produced in higher quantities than some androgens such as testosterone following puberty [9], and individuals with greater rates of steroid hormone synthesis may require a greater degree of enterohepatic disruption to achieve measurable changes in plasma hormone levels; this is an area that would benefit from additional investigation. Regardless, the dose and duration of BC and AC used in this study were sufficient to reduce fat androstenone levels; however, the sample size was limited, as a significant number of animals did not develop boar taint. Therefore, future research with larger sample sizes should evaluate lower BC inclusion levels and shorter treatment durations to optimize efficacy while reducing costs for producers.

The gut microbiome also plays a key role in both the EHC and circulating steroid hormone levels. The deconjugation of steroids by gut microbes is necessary for their reabsorption. *Firmicutes*, particularly members of the *Clostridia* class and *Ruminococcaceae* family, possess beta-glucuronidase activity to facilitate deconjugation [36], and their abundance was reported to correlate with non-ovarian estrogen levels in the systemic circulation [6,37]. Therefore, additional research is needed to investigate the relationship between the gut microbiome, boar taint status, and response to dietary BC treatment.

Due to their non-specific binding properties, AC and BC can bind many compounds in the intestine, including nutrients and hormones unrelated to boar taint that promote growth and development [14]. Therefore, we evaluated animal performance parameters in control compared to charcoal-treated boars throughout the trial. There were no differences in ADG or FCR over the entire trial duration between animals that received a charcoal-based treatment diet and those that received the control diet. Since it is possible that animals made up for any weight discrepancies during the acclimation or recovery periods, ADG and FCR were also evaluated in the treatment period exclusively. Again, there were no differences between animals on the control and charcoal-based treatment diets. It is possible that the consistent growth performance between treatment and control animals may be related to the similar plasma E1S concentrations between groups. These findings are similar to those previously reported by Schubert et al., [38], who tested coated and uncoated BC as a dietary method to prevent skatole accumulation in the fat of finisher boars. In their study, animals were fed a control diet or a BC-supplemented diet for fifteen or twenty-nine days. Regardless of the treatment duration, there were no differences in ADG or feed-to-gain ratios between the treatment and control boars. These results indicate that while AC and BC have the potential to bind nutrients as well as harmful compounds such as mycotoxins in the intestine [39], both growth rate and feed conversion efficiency were unaffected in this study. However, an important limitation to consider is that ADG and FCR were assessed at the pen level, not on an individual animal basis, which could reduce sensitivity in detecting treatment effects.

## 5. Conclusions

This study aimed to identify an alternative adsorbent to AC for the dietary prevention of boar taint. The in vitro binding kinetics of BC were most comparable to AC. Compared to SFA, both AC and BC had a higher binding affinity for androstenone, skatole, E1, and E1S. The AC also had the highest binding efficiency of the three adsorbents tested, indicating that higher concentrations of BC may be needed to achieve the same effects as AC. In subsequent in vivo feeding trials, both AC- and BC-supplemented diets kept fat androstenone levels below 1 µg/g in a subset of animals that responded to the treatments. Additionally, there were no differences in plasma androstenone or E1S levels among animals fed control, BC, or AC diets, and no negative effects on growth performance were observed from either charcoal-based treatment. Taken together, these results suggest that BC is a suitable alternative to AC and a promising dietary treatment for controlling boar taint in a subset of animals. However, additional work is needed to establish guidelines for optimal dosage and feeding duration before commercial use. There is also a need to develop genetic and biological markers that can accurately predict which animals both require treatment and are likely to respond effectively to BC, in order to use it as a treatment for controlling boar taint.

## Figures and Tables

**Table 1:** Diet compositions of trial diets including control, activated charcoal and biochar diets. Experimental diets contained a 5% inclusion of cellulose, activated charcoal, and biochar, respectively.

Ingredient Inclusion		Experimental Diets	
(g/kg)	Control	Activated Charcoal	Biochar
Corn	485	485	485
Soybean meal ^1^	200	200	200
Corn distillers dried grains and solubles	200	200	200
Corn oil	37.5	37.5	37.5
Vitamin/mineral premix ^2^	6	6	6
Mono-calcium phosphate	6.5	6.5	6.5
Limestone	9	9	9
Sodium chloride	3	3	3
L-Lysine-HCl	2.7	2.7	2.7
L-Threonine	0.3	0.3	0.3
Cellulose	50	-	-
Activated charcoal	-	50	-
Biochar	-	-	50
Total	1000	1000	1000

^1^ Soybean meal was dehulled and solvent extracted. ^2^ Vitamin/mineral premix used was swine vitamin tm micro premix produced by Grand Valley Fortifiers (Cambridge, ON, Canada).

**Table 2:** Binding kinetics data for steroids (E1, E1S, AND) (*n* = 3) and skatole (*n* = 2) binding agents using the Michaelis-Menten model.

Parameters		Activated Charcoal	Biochar	Spent Filter Aid
	*B_max_* (%)	98.3 ± 0.5 ^a^	99.7 ± 1.2 ^a^	39.8 ± 1.4 ^b^
E1	*K* (µg/mL)	25.4 ± 1.1 ^a^	72.0 ± 3.1 ^a^	423.3 ± 71.1 ^b^
	*B_max_/K* (% mL/mg)	3880.9 ± 163.1 ^a^	1390.1 ± 52.6 ^b^	99.6 ± 16.0 ^c^
	*B_max_* (%)	107.7 ± 0.4 ^a^	116.9 ± 0.3 ^b^	7.5 ± 0.8 ^c^
E1S	*K* (µg/mL)	38.1 ± 1.1 ^a^	128.5 ± 3.5 ^ab^	225.0 ± 58.3 ^b^
	*B_max_/K* (% mL/mg)	2891.8 ± 69.7 ^a^	910.8 ± 23.6 ^b^	34.1 ± 23.3 ^c^
	*B_max_* (%)	97.2 ± 0.4 ^a^	84.5 ± 0.8 ^b^	50.5 ± 0.2 ^c^
AND	*K* (µg/mL)	7.3 ± 0.2 ^a^	11.2 ± 0.5 ^a^	123.4 ± 7.9 ^b^
	*B_max_/K* (% mL/mg)	13,404.8 ± 334.3 ^a^	7553.1 ± 252.5 ^b^	412.5 ± 24.9 ^c^
	*B_max_* (%)	106.1 ± 0.2 ^a^	113.2 ± 0.7 ^a^	97.1 ± 5.3 ^b^
Skatole	*K* (µg/mL)	16.7 ± 0.9 ^a^	72.6 ± 4.6 ^a^	11,320.2 ± 1187.6 ^b^
	*B_max_/K* (% mL/mg)	6387.0 ± 325.2 ^a^	1383.3 ± 88.5 ^b^	8.6 ± 0.4 ^c^

E1 (estrone), E1S (estrone-1-sulfate), AND (androstenone), *B_max_* (maximum binding percentage), *K* (concentration of binding agent to bind 50% *B_max_*) and *B_max_/K* (binding efficiency). Different superscript letters within each row denote significant differences (*p* < 0.05) between adsorbents. Data are presented as means ± standard error.

**Table 3:** Comparison of ADG (average daily gain), FCR (feed conversion ratio) and weight of boars that received differing dietary treatments and multiple time points throughout the trial.

Performance Parameter	Control	Activated Charcoal	Biochar	*p*-Value
ADG over trial (kg/day)	1.37 ± 0.042	1.41 ± 0.045	1.39 ± 0.035	0.8
ADG during treatment (kg/day)	1.33 ± 0.050	1.28 ± 0.054	1.31 ± 0.050	0.6
FCR over trial	2.60 ± 0.052	2.56 ± 0.056	2.65 ± 0.088	0.06
FCR during treatment	2.60 ± 0.062	2.62 ± 0.076	2.74 ± 0.11	0.3
Weight at start of acclimation (kg)	70.98 ± 1.92	71.21 ± 2.59	71.71 ± 2.09	0.8
Weight at start of treatment (kg)	80.85 ± 2.57	82.60 ± 3.09	82.71 ± 3.07	0.8
Weight at end of treatment (kg)	125.06 ± 4.33	125.47 ± 4.20	125.74 ± 4.14	0.9
Weight at end of recovery (kg)	143.31 ± 4.92	145.68 ± 4.88	144.58 ± 4.72	0.9

ADG is calculated per animal, and FCR is calculated per pen. Data are presented as means ± standard error. *p*-values indicate significance (*p* < 0.05) across each row.

## Data Availability

The original contributions presented in the study are included in the article, further inquiries can be directed to the corresponding author.

## Supplementary Materials

The following supporting information can be downloaded at: https://www.mdpi.com/article/10.3390/biom15091257/s1, Table S1. Analysis of fat and plasma androstenone, and plasma E1S of all dietary treatment groups (control, AC, and BC), and animals grouped by treatment response (responded to treatment, did not respond to treatment, no boar taint development) at all time points throughout the trial.

## Abbreviations

The following abbreviations are used in this manuscript:

AC	Activated charcoal
ADG	Average daily gain
AND	Androstenone
BC	Biochar
CTRL	Control
EHC	Enterohepatic circulation
ELISA	Enzyme-Linked Immunosorbent Assay
E1	Estrone
E1S	Estrone-1-sulfate
FCR	Feed conversion ratio
PBS	Phosphate-buffered saline
RIA	Radioimmunoassay
SFA	Spent filter aid
