# Supplementary material for: In Vitro and In Vivo Comparisons of Activated Charcoal and Biochar as Dietary Treatments for Controlling Boar Taint
Source: Biomolecules. 2025 Aug 30;15(9):1257. doi: 10.3390/biom15091257 (PMC12467611; doi:10.3390/biom15091257)
Supplement: Supplementary file 1 [file biomolecules-15-01257-s001.zip › biomolecules-3801639-supplementary.pdf]

Supplementary Material

Supplementary Table S1. Analysis of fat and plasma androstenone, and plasma E1S of all dietary treatment groups (control, AC, and BC), and animals grouped by treatment response (responded to treatment, did not respond to treatment, no boar taint development) at all time points throughout the trial.

Boars Grouped by Diet or Treatment Response		n - value	Day of Trial							
			Day -7	Day 0	Day 7	Day 14	Day 21	Day 28	Day 35	Day 42
**Fat Androstenone (µg/g)**										
Diet	Total Control	16	-	0.27 ± 0.047[a]	-	0.47 ± 0.081[a]	-	0.79 ± 0.11[b1]	-	1.47 ± 0.28[b]
	Total AC	17	-	0.23 ± 0.027[a]	-	0.37 ± 0.043[ab]	-	0.54 ± 0.10[bc2]	-	1.35 ± 0.41[c]
	Total BC	17	-	0.25 ± 0.025[a]	-	0.43 ± 0.1[ab]	-	0.55 ± 0.075[b2]	-	1.34 ± 0.32[c]
Treatment Response	Responded	15	-	0.234 ± 0.027[a]	-	0.41 ± 0.056[ab]	-	0.59 ± 0.052[b2]	-	1.69 ± 0.18[c1]
	Did Not Respond	3	-	0.29 ± 0.11[a]	-	0.60 ± 0.24[a]	-	1.24 ± 0.13[b1]	-	3.43 ± 0.71[c2]
	No Boar taint	26	-	0.25 ± 0.036[a]	-	0.36 ± 0.035[a]	-	0.40 ± 0.038[a2]	-	0.86 ± 0.051[b3]
**Plasma Androstenone (ng/mL)**										
Diet	Total Control	16	37.13 ± 4.95[a]	44.99 ± 5.80[a]	39.61 ± 3.71[a]	46.20 ± 4.68[a]	41.53 ± 6.44[a]	54.52 ± 5.95[a]	58.06 ± 5.60[a]	85.06 ± 7.23[a]
	Total AC	17	35.50 ± 5.22[a]	39.53 ± 4.14[a]	35.85 ± 5.05[ab]	42.03 ± 5.94[ab]	30.05 ± 4.03[ab]	46.30 ± 6.42[ab]	47.96 ± 4.21[ab]	71.96 ± 7.93[b]
	Total BC	17	27.44 ± 3.86[a]	35.04 ± 5.63[a]	39.31 ± 5.77[a]	37.87 ± 5.53[a]	41.23 ± 5.97[a]	55.31 ± 5.21[a]	45.83 ± 5.56[a]	64.67 ± 8.54[a]
Treatment Response	Responded	15	42.44 ± 8.91[a]	49.55 ± 8.80[a]	43.08 ± 5.79[a]	50.80 ± 8.11[a]	56.03 ± 12.22[a]	62.79 ± 10.26[a]	60.43 ± 6.80[a]	94.60 ± 10.02[b]
	Did Not Respond	3	28.89 ± 6.53[a]	36.34 ± 6.53[a]	31.24 ± 4.30[a]	39.05 ± 7.78[a]	34.37 ± 7.63[a]	47.65 ± 9.82[a]	53.78 ± 4.78[a]	91.58 ± 11.95[a]
	No Boar taint	26	34.48 ± 5.70[a]	42.93 ± 8.31[ab]	37.84 ± 8.73[ab]	37.88 ± 8.57[ab]	44.26 ± 10.18[ab]	51.88 ± 9.57[bc]	52.03 ± 9.47[bc]	78.53 ± 15.11[c]
**Plasma E1S (ng/mL)**										
Diet	Total Control	16	5.87 ± 1.50[a]	9.76 ± 1.86[be]	12.07 ± 2.36[bce]	13.33 ± 2.79[abde]	15.41 ± 3.16[abf]	24.60 ± 5.36[eg]	26.75 ± 5.67[cdfg]	25.82 ± 3.04[eg]
	Total AC	17	6.22 ± 1.71[a]	9.96 ± 2.14[a]	7.93 ± 1.71[a]	10.37 ± 1.77[a]	11.27 ± 2.21[a]	16.47 ± 3.26[a]	20.07 ± 4.00[a]	22.79 ± 4.37[a]
	Total BC	17	4.48 ± 0.86[a]	6.26 ± 1.20[a]	7.37 ± 1.48[a]	8.13 ± 1.27[ab]	12.41 ± 2.63[ab]	15.89 ± 2.94[ab]	19.01 ± 3.80[ab]	25.09 ± 3.77[b]
Treatment Response	Responded	15	7.03 ± 2.78[a]	11.58 ± 3.12[ab]	14.84 ± 4.83[abc]	15.20 ± 6.11[abc]	17.55 ± 6.69[bc]	33.14 ± 12.12[cd]	40.83 ± 12.97[d1]	30.72 ± 6.12[d12]
	Did Not Respond	3	11.29 ± 3.38[a]	15.06 ± 4.40[a]	13.30 ± 3.06[a]	14.90 ± 3.11[a]	18.79 ± 3.38[ab]	22.04 ± 5.37[bc]	31.75 ± 7.20[bc1]	26.87 ± 5.68[c1]
	No Boar taint	26	5.19 ± 1.28[a]	5.44 ± 1.40[ab]	8.98 ± 2.17[ab]	10.13 ± 2.16[ab]	15.37 ± 4.04[ab]	18.34 ± 4.03[b]	22.43 ± 4.58[b2]	25.85 ± 4.79[c2]

E1S (estrone-1-sulfate), AC (activated charcoal), BC (biochar). Treatment response excludes control boars that developed boar taint (n=6). Data are presented as means ± standard error. Different superscript letters across rows denote significant differences ($p<0.05$) over time, and different superscript numbers down columns denote significant differences ($p<0.05$) between diets or boar taint phenotype groups.
